# Ophthalmology

**Published:** 1898-10

**Authors:** Alvin A. Hubbell

**Affiliations:** Buffalo, N. Y.; Clinical professor of ophthalmology and otology at the University of Buffalo


### OPHTHALMOLOGY.

CONDUCTED BY ALVIN A. HUBBELL, M. D., Buffalo, N. Y.
Clinical professor of ophthalmology and otology at the University of Buffalo.

### NOTE ON PROTARGOL IN OPHTHALMIC PRACTICE.

DR. ADOLF ALT (*American Journal of Ophthalmology*, January, 1898,) says according to Neisser: " The new silver-salt, protargol, is prepared by Fr. Bayer & Co., in Elberfeld, Germany, and contains 8.3 per cent. of silver. It is a chemical combination of silver with a protein substance, and forms a yellowish fine powder which can be solved in cold or hot water by shaking. Heating to a high degree does not render the solution dim. Its most important peculiarity, not shared by any other silver salt, is that from the aqueous solution it is not precipitated by either albumin, diluted chlorate of sodium, diluted muriatic acid or caustic soda. Ammonium sulphate gives it a darker color, but causes no precipitate. Concentrated muriatic acid produces a precipitate, yet what is precipitated is not silver chloride, but protargol, which, when more water is added, is redissolved.

"This salt has as great a faculty of an unlimited penetrating action on the tissues as no other silver salt enjoys. It is superior to argentamin which, otherwise, is the best silver-salt, in that in solutions of one-fourth, one half and 1 per cent. it causes but an extremely small amount of irritation. The experiments concerning the bactericidal action of the protargol are as yet unfinished. Its action on the mucous membrane may be prolonged without a weakening of its concentration or diminution of its action on the deep tissues being produced by any chemical changes."

Dr. Alt says the results which Neisser has reached by using this new silver-salt in treating inflammation of urethral mucous membrane and particularly the statement that it produces almost no irritation while being as effective and more so than silver-nitrate on account of its penetration into the depths of the tissues, prompted me to give protargol a trial in the treatment of conjunctival inflammation, in which I had thus far applied silver-nitrate.

While I am now, after two months' trial, not trying to praise protargol as a panacea for all conjunctival inflammations, I am so impressed and pleased with its beneficial and almost painless action on the conjunctiva, that I want to draw the attention of my confréres to this new silver-salt, protargol, as early as possible.

As far as my experience goes, it acts as well as silver-nitrate; sometimes, indeed, in cases of acute conjunctivitis I am satisfied it acted quicker than the older favorite.

I have used it thus far only in a 1 per cent. solution. In this strength it causes no noticeable inconvenience to the patients who, on that account, greatly prefer it to the silver-nitrate.

It would be tiresome to relate individual cases here. It may suffice to repeat, that wherever silver-nitrate is indicated, it is well to give protargol a trial instead, and I have no doubt that in a great many cases, if not in all, the results will be gratifying.

### CATARACT IN GLASSBLOWERS.

HIRSCHBERG (*Berl. Klin. Woch.*, February 7, 1898). The etiologic connection between cataract formation and occupations entailing exposure to intense heat, as well as residence in hot climates, has long been recognised. Of thirty employed as glassblowers, but five had reached the age of forty, and all of these had developed glass-blowers' cataract. The frequency of cataract in India is well known. The comparatively early age at which this affection develops, as the result of exposure to heat, has never before been dwelt upon. Whereas in senile cataract the average age is in the neighborhood of sixty-six years, in glassblowers' cataract and among the inhabitants of India the average is forty years.

---

### THE PUPIL IN DISEASE.

ACCORDING to Frank C. Todd, M. D., Minneapolis, Minn., (*Annals of Ophthalmology*, April, 1898,) irritation myosis is found in: (1) The early stages, at least, of all inflammatory affections of the brain and its meninges, in simple tubercular and cerebrospinal meningitis. When in these diseases medium myosis gives place to mydriasis, the change is a serious prognostic sign, indicating the stage of depression with paralysis of the third nerve. (2) In cerebral apoplexy the pupil is at first contracted, according to Berthold, who points out that this contraction is a diagnostic sign between apoplexy and embolism, in which latter the pupil is unaltered. (3) In the early stages of intracranial tumor, situated at the origin of the nerve (third) or in its course. (4) At the beginning of an hysterical or of ane pileptic attack. (5) In tobacco amblyopia, probably from stimulation by nicotine. (6) In persons following certain trades, as a result of long maintained effort of accommodation (watchmakers and the like) the pupil-contracting center being subject to an almost constant stimulus. (7) As a reflex action in ciliary neurosis; consequently, in many diseased conditions of those parts of the eye supplied by the fifth nerve. (8) In iritis.

Paralytic myosis occurs: (1) In spinal lesions above the dorsal region, *e. g.*, injuries and inflammations, especially of the chronic form. (2) In general paralysis of the insane. In acute mania the pupil is usually much dilated and when myosis develops, approaching general paralysis may be prognosticated. (3) In myelitis of the cervical portion of the cord, following irritation mydriasis. (4) In bulbar paralysis, with progressive muscular atrophy or sclerosis of the brain and spinal cord. (5) In alcoholic amblyopia. (6) In paralysis of the cervical sympathetic, resulting from injury, from pressure of an aneurism of the carotid, innominate or aorta, or from pressure of enlarged lymphatic glands. (7) In apoplexy of the pons Varolii this may be irritation myosis. (8) In poisoning by certain drugs known as myotics.

Irritation mydriasis occurs: (1) In hyperemia of the cervical portion of the spinal cord and in spinal meningitis. (2) In the early stages of new growths in the cervical portion of the cord. (3) In cases of intracranial tumors and other diseases causing high intracranial pressure—these may cause paralytic mydriasis. (4) In the spinal irritation of chlorotic or anemic people after severe illness, and the like. (5) As a premonitory sign of tabes dorsalis. (6) In cases of intestinal worms and other forms of intestinal irritation. (7) In psychical excitement, such as acute mania, melancholia, progressive paralysis of the insane—may be myosis on one side, mydriasis on the other.

Paralytic mydriasis occurs: (1) In progressive paralysis following myosis. (2) In various disease processes, such as syphilis at the base of the brain, affecting the third nerve. (3) In a late stage of thrombosis of the cavernous sinus. (4) In orbital processes, which cause pressure on the ciliary nerves. (5) In glaucoma. (6) In cases of large intraocular tumors. (7) In cases of poisoning by alkaloids, known as mydriatics and by toxic principles of putrefaction —rotten meat, and the like. (8) Following diphtheria. (9) Following a traumatism to the eye-ball.

The Argyll-Robertson pupil is one that reacts to convergence, but not to light and indicates a lesion of the centripetal fibers, in the tabes dorsalis, probably Meynert's.

Reaction of the pupil in hemianopia is of importance in diagnosticating the location of a lesion in the brain. A lesion is anterior to the primary optic centers in hemianopia, if myosis takes place when light is thrown upon the seeing side of the retina, and does not take place when thrown upon the blind side. A lesion is posterior to the primary optic centers when myosis occurs when the light is thrown upon either side of the retina.

---

### BACTERIA IN THE NORMAL CONJUNCTIVA, AND THE EFFECTS UPON THEM OF ASEPTIC AND ANTISEPTIC IRRIGATIONS.

DR. RANDOLPH, of Baltimore, Md., (*Journal of the Amer. Med. Assn.*, January 8, 1898,) has made a series of experiments upon one hundred persons (not eye patients) using the conjunctival secretion of these persons for inoculation on nutrient agar. Out of the one hundred cases there were only thirteen sterile tubes. No effort was made to determine the properties of the microörganism.

Two series of experiments of fifty each were made, one to test the aseptic and the other the antiseptic method of sterilising the field of operation. After repeated irrigation of the conjunctiva with sterilised water, inoculations showed a growth in thirty-two out of forty tubes. After flooding with a solution of bichloride 1 to 5,000 and inoculation with conjunctival secretions there were only nine sterile tubes. These experiments showed that, as far as the conjunctiva is concerned, little reliance can be placed on the usual methods employed by ophthalmologists for obtaining asepsis. Dr. Randolph adds the following conclusions :

" The normal conjunctiva always contains bacteria. It is probable that the bacteria found in this locality are usually of only slight, if any, pathogenic character. It should be remembered, though, that bacteria, ordinarily nonpathogenic, may become harmful under certain conditions ; that is, if the tissues are bruised by instruments or irritated by chemicals. Neither the irrigation with sterilised water, nor the instillation of a sublimate solution (1 to 5,000) produces sterility of the conjunctiva and inasmuch as both measures are futile and possibly harmful, they may just as well be abandoned. These methods of sterilising the conjunctiva are the only ones usually employed by ophthalmologists, and hence the choice of them for testing this question. It goes without saying that a method which would destroy the bacteria of the conjunctiva without at the same time impairing the integrity of this membrane would be a great advantage. In operating upon the normal conjunctiva, as in cataract operations, the surgeon in the present state of our disinfecting armamentarium would do well to consider the subject of antiseptics chiefly, if not solely, in connection with his hands and instruments, and, of course, the cocain and atropin."

---

THE BANANA IN TYPHOID FEVER.—After a long experience with typhoid patients, Dr. Ussery, of St. Louis, regards the banana as the best food for them. A banana is almost wholly absorbed by the stomach, easily digested and very strengthening.—*Medical Age.*

